# CrBPF1 overexpression alters transcript levels of terpenoid indole alkaloid biosynthetic and regulatory genes

**DOI:** 10.3389/fpls.2015.00818

**Published:** 2015-10-01

**Authors:** Chun Yao Li, Alex L. Leopold, Guy W. Sander, Jacqueline V. Shanks, Le Zhao, Susan I. Gibson

**Affiliations:** ^1^Department of Plant Biology, University of Minnesota Twin Cities, Saint PaulMN, USA; ^2^Department of Chemical Engineering, University of Minnesota DuluthDuluth, MN, USA; ^3^Department of Chemical and Biological Engineering, Iowa State UniversityAmes, IA, USA

**Keywords:** *Catharanthus roseus*, CrBPF1, GBF, ORCA2, ORCA3, terpenoid indole alkaloid, transgenic hairy roots, ZCT

## Abstract

Terpenoid indole alkaloid (TIA) biosynthesis in *Catharanthus roseus* is a complex and highly regulated process. Understanding the biochemistry and regulation of the TIA pathway is of particular interest as it may allow the engineering of plants to accumulate higher levels of pharmaceutically important alkaloids. Toward this end, we generated a transgenic *C. roseus* hairy root line that overexpresses the *CrBPF1* transcriptional activator under the control of a β-estradiol inducible promoter. CrBPF1 is a MYB-like protein that was previously postulated to help regulate the expression of the TIA biosynthetic gene *STR*. However, the role of CrBPF1 in regulation of the TIA and related pathways had not been previously characterized. In this study, transcriptional profiling revealed that overexpression of *CrBPF1* results in increased transcript levels for genes from both the indole and terpenoid biosynthetic pathways that provide precursors for TIA biosynthesis, as well as for genes in the TIA biosynthetic pathway. In addition, overexpression of *CrBPF1* causes increases in the transcript levels for 11 out of 13 genes postulated to act as transcriptional regulators of genes from the TIA and TIA feeder pathways. Interestingly, overexpression of *CrBPF1* causes increased transcript levels for both TIA transcriptional activators and repressors. Despite the fact that *CrBPF1* overexpression affects transcript levels of a large percentage of TIA biosynthetic and regulatory genes, *CrBPF1* overexpression has only very modest effects on the levels of the TIA metabolites analyzed. This finding may be due, at least in part, to the up-regulation of both transcriptional activators and repressors in response to *CrBPF1* overexpression, suggesting that CrBPF1 may serve as a “fine-tune” regulator for TIA biosynthesis, acting to help regulate the timing and amplitude of TIA gene expression.

## Introduction

Madagascar periwinkle *Catharanthus roseus* (L.) G. Don is of substantial pharmaceutical interest as it produces over 130 terpenoid indole alkaloids (TIAs). Several of these TIAs are used to treat different medical conditions. For example, vinblastine and vincristine are widely used as anticancer agents in the treatment of lymphoma and leukemia (Gidding et al., 1999) and ajmalicine and serpentine may be used to treat hypertension. Unfortunately these plant-derived pharmaceutical compounds are very expensive as periwinkle produces TIAs in very low amounts. In addition, due to their complex chemical structures, TIAs tend to be difficult, and therefore expensive, to synthesize *in vitro*. To lower the costs of producing TIAs for use as pharmaceuticals, many efforts have been made to increase TIA production using plant tissue and cell cultures or bacterial cultures. However, despite efforts since the early 1980s to develop cost-effective methods for large-scale production of TIAs in cultures or *in vitro*, progress has been limited (van der Heijden et al., 2004; Shanks, 2005).

**FIGURE 1:**
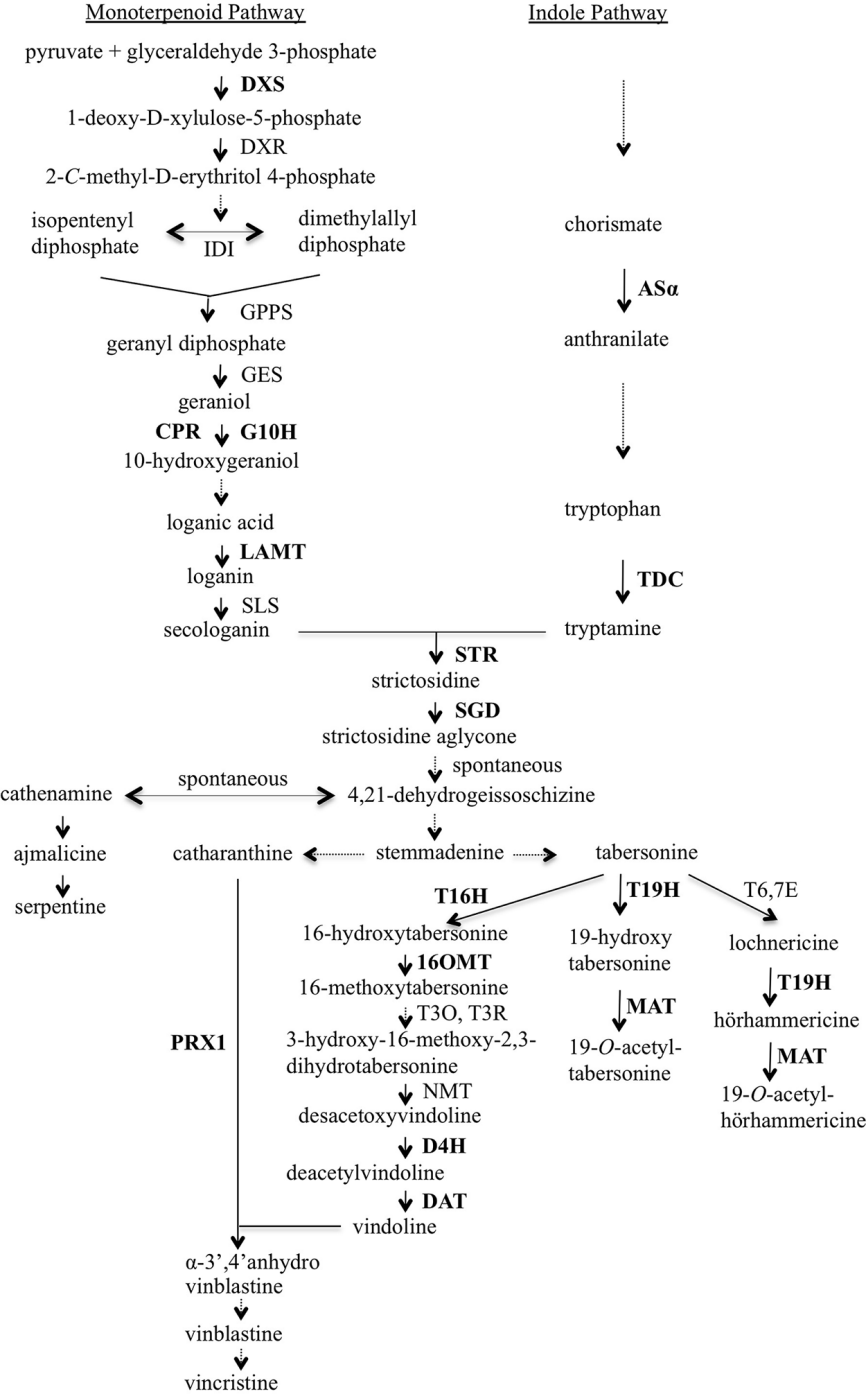
**Overview of terpenoid indole alkaloid (TIA) biosynthesis in *Catharanthus roseus***. Enzymes are indicated using capital letters and metabolites using lowercase letters. Solid arrows indicate single enzymatic conversions; dashed arrows indicate multiple enzymatic reactions. The genes analyzed in this work are shown in bold type. 16OMT, 16-hydroxytabersonine-*O*-methyl-transferase; 19H, 19-hydroxylase; AS, anthranilate synthase; CPR, cytochrome P450 reductase; D4H, desacetoxyvindoline 4-hydroxylase; DAT, deacetylvindoline 4-*O*-acetyltransferase; DXR, 1-deoxy-D-xylulose-5-phosphate reductoisomerase; DXS, 1-deoxy-D-xylulose-5-phosphate synthase; G10H, geraniol 10-hydroxylase; GES, geraniol synthase; GPPS, geranyl diphosphate synthase; IDI, isopentenyl diphosphate isomerase; LAMT, loganic acid *O*-methyltransferase; MAT, minovincinine-19-*O*-acetyltransferase; NMT, *N*-methyltransferase; PRX1, vacuolar class III peroxidase; SGD, strictosidine glucosidase; SLS, secologanin synthase; STR, strictosidine synthase; T3O, tabersonine 3-oxygenase; T3R, tabersonine 3-reductase; T6,7E, tabersonine 6,7-epoxidase; T16H, tabersonine 16-hydroxylase; T19H, tabersonine/lochnericine19-hydroxylase; TDC, tryptophan decarboxylase.

A major reason why these efforts have not been more successful is that the TIA biosynthetic pathway and the regulation of the TIA pathway and TIA feeder pathways are not sufficiently well understood. Those deficiencies are beginning to be addressed, thanks to progress in identifying most of the genes encoding TIA biosynthetic enzymes and transcriptional regulators (Liu et al., 2007; Memelink and Gantet, 2007; Zhou et al., 2010a; Giddings et al., 2011; Asada et al., 2013; Besseau et al., 2013; Salim et al., 2013; Zhao et al., 2013; De Luca et al., 2014; Kellner et al., 2015; Qu et al., 2015; Van Moerkercke et al., 2015). TIA biosynthesis is a closely coordinated process involving many enzymatic steps that occur in several intra- and inter-cellular compartments (Burlat et al., 2004; van der Heijden et al., 2004; Murata et al., 2008; De Luca et al., 2014; Qu et al., 2015). TIA biosynthesis in *C. roseus* starts with the formation of strictosidine from tryptamine and secologanin, the two precursor molecules that are produced by the indole and the monoterpenoid pathways, respectively (**Figure 1**). The condensation process is catalyzed by strictosidine synthase (STR). Strictosidine is then deglucosylated by strictosidine β-D-glucosidase (SGD) to form strictosidine aglycone. Further enzymatic steps result in the formation of numerous TIAs via several specific branches. For example, one branch of the TIA pathway produces ajmalicine and serpentine, a second branch leads to production of lochnericine and hörhammericine, a third branch produces vindoline and a fourth branch produces catharanthine. The production of vindoline from tabersonine occurs via seven reactions. Genes encoding enzymes catalyzing all seven steps have now been identified (Vazquez-Flota et al., 1997; St-Pierre et al., 1998; Schröder et al., 1999; Levac et al., 2008; Liscombe et al., 2010; Qu et al., 2015). Vindoline and catharanthine are the substrates for a major class III peroxidase (PRX1), which catalyzes formation of α-3′,4′-anhydrovinblastine (Costa et al., 2008). Vinblastine and vincristine, two of the most pharmaceutically important TIAs, are formed through multiple steps from α-3′,4′-anhydrovinblastine.

Transcriptional activators and repressors play important roles in regulating TIA biosynthesis. Expression of both Octadecanoid-Responsive Catharanthus AP2-domain protein 2 (ORCA2) and ORCA3 increases rapidly upon fungal elicitation (Menke et al., 1999; van der Fits and Memelink, 2000). ORCA2 and ORCA3 are AP2-domain transcription factors that activate *STR* expression through a jasmonate signal transduction pathway by binding to the jasmonate and elicitor-responsive element (JERE) in the *STR* promoter (Menke et al., 1999; van der Fits and Memelink, 2001). ORCA2 (Li et al., 2013) and ORCA3 (van der Fits and Memelink, 2000; Peebles et al., 2009; Wang et al., 2010; Zhou et al., 2010b; Tang et al., 2011; Pan et al., 2012; Van Moerkercke et al., 2015) have also been shown to regulate many additional TIA biosynthetic and regulatory genes. The levels of some TIAs are also affected by overexpression of ORCA2 (Liu et al., 2011; Li et al., 2013) and/or ORCA3 (van der Fits and Memelink, 2000; Peebles et al., 2009; Wang et al., 2010; Zhou et al., 2010b; Tang et al., 2011; Pan et al., 2012). BIS1, a basic Helix-Loop-Helix transcription factor, regulates expression of the genes encoding all of the enzymes necessary for the conversion of geranyl diphosphate to loganic acid (Van Moerkercke et al., 2015). The CrMYC2 (basic Helix-Loop-Helix) transcription factor helps regulate TIA production by controlling the jasmonate-responsive expression of the *ORCA* genes (Zhang et al., 2011). Two AT-hook DNA binding proteins, 2D173 and 2D7, also help regulate *ORCA3* expression (Vom Endt et al., 2007). The CrMYC1 transcription factor helps regulate *STR* expression and is responsive to both jasmonate and elicitor treatments (Chatel et al., 2003). Similarly, the CrWRKY1 and CrWRKY2 transcription factors exert positive regulatory effects on multiple TIA biosynthetic genes (Suttipanta, 2011; Suttipanta et al., 2011). However, overexpression of *CrWRKY1* and *CrWRKY2* has contrasting effects on expression of specific TIA regulatory genes. Overexpression of *CrWRKY1* results in decreased transcript levels for the *ORCA2*, *ORCA3*, and *CrMYC2* TIA transcriptional activators and increased transcript levels for the *ZCT1*, *ZCT2*, and *ZCT3* TIA transcriptional repressors (Suttipanta, 2011), whereas overexpression of *CrWRKY2* results in increased transcript levels for both the *ORCA2*, *ORCA3*, and *CrWRKY1* TIA transcriptional activators and the *ZCT1* and *ZCT3* TIA transcriptional repressors (Suttipanta et al., 2011). In addition to transcriptional activators, three zinc finger proteins, ZCT1, ZCT2, and ZCT3 (Pauw et al., 2004; Chebbi et al., 2014) and two G-box-binding factors, GBF1 and GBF2 (Sibéril et al., 2001) act as transcriptional repressors of specific genes from the TIA or TIA feeder pathways.

CrBPF1 was identified as a protein that binds an element in the *C. roseus STR* promoter that is distinct from the element bound by the ORCAs (van der Fits et al., 2000). CrBPF1 contains a single MYB-like DNA binding domain near its C-terminus. The presence of a single MYB-like sequence places CrBPF1 in the R-MYB family of proteins (Feller et al., 2011). Sequence analysis showed that CrBPF1 has high homology to the parsley box P-binding factor-1 (BPF-1; da Costa e Silva et al., 1993), which accumulates rapidly in elicitor-treated parsley cells and around fungal infection sites on parsley leaves. A strong correlation between *BPF-1* transcript levels and *phenylalanine ammonia-lyase* (*PAL*) expression in parsley cell cultures suggests that BPF-1 might play an important role in disease resistance by helping regulate expression of plant defense genes (da Costa e Silva et al., 1993). In *C. roseus* CrBPF1 was found to bind specifically to the BA element within the *STR* promoter. CrBPF1 promotes *STR* transcription through a signal transduction pathway that is responsive to elicitors but not jasmonate and acts downstream of protein phosphorylation and calcium influx (van der Fits et al., 2000). However, current evidence indicates that CrBFP1 activity is not sufficient for elicitor-induced STR expression (van der Fits et al., 2000). Deletion of the BA fragment did not eliminate the ability of the STR promoter to respond to elicitor or jasmonate, whereas alteration or deletion of the JERE fragment rendered the STR promoter unable to respond to either of these compounds (Menke et al., 1999).

Information regarding whether CrBPF1 plays a role in the regulation of TIA-related genes other than *STR* has not previously been reported, leaving the role of CrBPF1 in regulation of TIA metabolism unknown. To address this issue, a transgenic hairy root line of *C. roseus* that overexpresses *CrBPF1* under the control of a β-estradiol inducible promoter was generated. The transcript levels of 31 TIA biosynthetic and regulatory genes were tracked over a 72-h period under β-estradiol-induced and un-induced condition. The levels of 14 metabolites from the TIA and TIA feeder pathways were also investigated over the same time course, with nine of those metabolites being present at detectable levels in the majority of the samples analyzed. The results of these transcriptional and metabolic profiling experiments have revealed the role of CrBPF1 in regulation of TIA metabolism.

## Materials and Methods

### Plant Materials and Growth Conditions

*Catharanthus roseus*, Vinca Little Bright Eye^1^, was used for this work. Seeds were surface sterilized and then germinated on B5 medium (Sigma, St. Louis, MO, USA) supplemented with Gamborg’s vitamins (Sigma, St. Louis, MO, USA). Seeds were germinated in the dark at 26°C for 2 weeks. The seedlings were then transferred to a 16-h-light/8-h-dark cycle with a light intensity of approximately 44 μmol m^-2^ s^-1^ for 4 weeks before inoculation with *Agrobacterium tumefaciens*.

### Generation of *CrBPF1* Overexpression Construct

The pERKT vector (Tsuda et al., 2012) is a modified version of the pMDC32 Gateway vector (Curtis and Grossniklaus, 2003) that expresses the XVE chimeric transcriptional activator (Zuo et al., 2000) under the control of a strong constitutive promoter. XVE allows estradiol-inducible expression of genes cloned behind an appropriate promoter sequence (Zuo et al., 2000). For this work, the full-length *CrBPF1* open reading frame was inserted into pERKT behind a promoter that allows estradiol-inducible expression by XVE. DNA containing the *CrBPF1* open reading was obtained by PCR amplification of *C. roseus* cDNAs using KOD Hot Start DNA polymerase (Novagen, Madison, WI, USA) and the following primer pair: 5′ ATGGTGTTGAAGAGAAGGC 3′ and 5′ TTAATCCGCCTGAGCATCC 3′. The resulting PCR fragment was cloned into the pCR8/GW/TOPO entry vector (Invitrogen, Grand Island, NY, USA) and then transferred to pERKT by an LR reaction to form pERKT-CrBPF1 (Supplemental Figure S1).

### Generation of Transgenic *C. roseus* Hairy Roots

Transformation of 6-weeks old *C. roseus* was carried out using an equal mixture of *A. tumefaciens* cultures transformed with pERKT-CrBPF1 or the pPZPROL plasmid that carries the *rol ABC* genes (Hong et al., 2006), as previously described (Hong et al., 2006). Hairy roots appeared on infection sites approximately 4 weeks after inoculation. These hairy roots were allowed to grow to a length of approximately 1 cm and then excised and transferred to solid medium supplemented with 30 g L^-1^ sucrose, 6 g L^-1^ agar, 250 mg L^-1^ cefotaxime, half-strength Gamborg’s B5 salts, and full-strength Gamborg’s vitamins (pH 5.8). One week after transfer to the above media, 30 mg L^-1^ hygromycin was used to select for roots carrying the pERKT-CrBPF1 construct. Hygromycin-resistant hairy roots were further screened by PCR using primers with sequences 5′ ATGATCACAAGCTGATCCCC 3′ and 5′ GTGCGTTCGGAAAAAGAATC 3′ to amplify a DNA sequence that spans *CrBPF1* and adjacent vector sequences within pERKT-CrBPF1. Transgenic hairy root lines exhibiting hygromycin-resistance and a positive reaction in the PCR test were transferred to 50 mL of liquid media containing half-strength Gamborg’s B5 liquid solution supplemented with full-strength Gamborg’s vitamins and 30 g L^-1^ sucrose in a 150-mL flask. Hairy root cultures were incubated in the dark on a shaker at 225 rpm and sub-cultured every 5 weeks.

### Induction of Transgene Expression and Tissue Collection

One transgenic hairy root line carrying the pERKT-CrBPF1 overexpression construct (CrBPF1-OE) and one control line carrying only the pPZPROL transformation construct (Li et al., 2013) were used for time course analyses. Each transgenic hairy root culture was started from five hairy roots that were 3–4 cm in length. These cultures were grown for 35 days and then transferred to fresh liquid media with 20-μM β-estradiol (induced cultures) or without β-estradiol (un-induced cultures) for 0, 6, 12, 24, 48, or 72 h before being harvested. Three separate cultures were harvested for each hairy root line, time point and treatment condition. Hairy roots were flash frozen immediately after harvest using liquid nitrogen and then stored at –80°C. Aliquots of the same tissue samples were used for analyses of both transcript and metabolite levels.

### RNA Extraction and qRT-PCR Analyses

Total RNA was isolated using the Spectrum Total RNA Isolation Kit with on-column DNase 1 digestion (Sigma, St. Louis, MO, USA), as described (Huang et al., 2010). For quantification of transcripts produced by *BIS1*, the endogenous and trans *CrBPF1* genes, *CrMYC1*, *CrMYC2*, *CrWRKY1*, *CrWRKY2*, *DXS1*, *DXS2B*, *MAT*, *T16H2*, and *T19H* the GoScript Reverse Transcription System (Promega, Madison, WI, USA) was used to produce cDNAs. These cDNAs were analyzed by qPCR using the LightCycler 480 SYBR Green I Master mix by Roche and a Roche LightCycler 480 II (Roche Diagnostics, Indianapolis, IN, USA). For quantification of transcripts produced by all other genes (including the total transcripts produced by the *CrBPF1* endogenous and transgenes combined), SuperScript II reverse transcriptase (Invitrogen, Grand Island, NY, USA) was used for cDNA synthesis and qPCR was performed on an ABI 7900 HT (Applied Biosystems, Grand Island, NY, USA) with a 384-well ABI optical plate using Roche Universal Probes (Roche Applied Science, USA) and the Homebrew master mix (University of Minnesota Genomics Center, Minneapolis, MN, USA). PCR primers and Roche Universal Probe numbers are described in Supplemental Table S1. qPCR data were normalized using the geometric average (Vandesompele et al., 2002) of two control genes, *EF1* and *UBQ11*, which exhibit particularly stable expression patterns in *C. roseus* (Wei, 2010). Differences in EF1 and UBQ11 ΔCT levels were typically minor (Supplemental Table S2). Relative mRNA levels for each gene were converted to ΔΔCt. ΔΔCt = ΔCt_un-induced_ _control_ _line_ _at__0__h_ - ΔCt_other_. ΔCt_un-induced_ _control_ _line_ _at__0__h_ = CT_indicated_ _gene_ _in_ _un-induced_ _control_ _line_ _at__0__h_ - CT*_EF1/UBQ11_*_in_ _un-induced_ _control_ _line_ _at__0__h_. ΔCt_other_ = CT_indicated_ _gene_ - CT*_EF1/UBQ11_* for the indicated line, growth condition, and time point. The lone exception is that mRNA levels for the *CrBPF1* transgene were normalized versus mRNA levels from the endogenous *CrBPF1* gene in the un-induced control line at 0 h rather than against *CrBPF1* transgene mRNA levels in the un-induced control line at 0 h because the control line lacks the *CrBPF1* transgene. A positive ΔΔCt value indicates that mRNA levels for the indicated gene are higher in the indicated hairy root line grown for the indicated time under the indicated conditions than in the un-induced control line at 0 h. Negative ΔΔCt values indicate the reverse situation. As the amount of PCR product approximately doubles with each reaction cycle, a ΔΔCt of one corresponds to approximately a twofold difference in transcript levels. The genes analyzed in this study are: *16OMT* [GenBank: EF444544], *AS*α [GenBank: AJ250008], *BIS1* [GenBank: KM409646], *CPR* [GenBank: X69791], *CrBPF1* [GenBank: AJ251686], *CrMYC1* [GenBank: AF283506], *CrMYC2* [GenBank: AF283507], *CrWRKY1* [GenBank: HQ646368], *CrWRKY2* [GenBank accession number not available], *D4H* [GenBank: U71605], *DAT* [GenBank: AF053307], *DXS1* [GenBank: KC625536], *DXS2A* [GenBank: AJ011840], *DXS2B* [GenBank: DQ8486762], *EF1* [GenBank: EU007436], *G10H* [GenBank: AJ251269], *GBF1* [GenBank: AF084971], *GBF2* [GenBank: AF084972], *LAMT* [GenBank: EU057974], *MAT* [GenBank: AF253415], *ORCA2* [GenBank: AJ238740], *ORCA3* [GenBank: EU072424], *PRX1* [GenBank: AM236087], *SGD* [GenBank: EU072423], *STR* [GenBank: X53602], *T16H1* [GenBank: FJ647194], *T16H2* [GenBank: JF742645], *T19H* [GenBank: HQ901597], *TDC* [GenBank: X67662], *UBQ11* [GenBank: EU007433], *ZCT1* [GenBank: AJ632082], *ZCT2* [GenBank: AJ632083], *ZCT3* [GenBank: AJ632084].

### Alkaloid Extraction and Analysis

Frozen hairy root tissue samples were lyophilized and ground to a fine powder. Fifty milligram aliquots of the ground tissue samples were extracted on ice for 10 min using 10 mL of methanol and a Model VC 130PB sonicating probe (Sonics & Materials, Inc., Newton, CT, USA), as previously described (Sander, 2009). The extracts were centrifuged for 12 min at 4000 rpm at 15°C. The biomass was re-extracted as above and the supernatants combined and passed through a 0.45 μm nylon filter (25 mm, PJ Cobert, St Louis, MO, USA). The supernatants were then dried using a nitrogen evaporator (Organomation Associates, Inc., Berlin, MA, USA). The residues were dissolved in 2 mL of methanol, filtered using a 0.22 μm nylon filter (13 mm, PJ Cobert) and then stored at –25°C.

Twenty-microliter aliquots of the extracted alkaloid concentrate were run on a Phenomenex Luna C18(2) column (250 mm × 4.6 mm) connected to a Waters high performance liquid chromatography system [1525 binary pump, 717plus Autosampler, 996 Photo Diode Array (PDA) detector] using three different solvent systems. TIAs were examined following a previously described method (Sander, 2009). PDA data extracted at 254 nm were compared to standards for the quantification of strictosidine (gift from Dr. O’Connor, John Innes Centre, UK), ajmalicine (Fluka/Sigma, St. Louis, MO, USA), serpentine (Sigma–Aldrich, St. Louis, MO, USA), vindoline (ChemPacific Corp., Baltimore, MD, USA), vincristine (Sigma, St. Louis, MO, USA), vinblastine (Sigma, St. Louis, MO, USA), and catharanthine (Qventas, Branford, CT, USA). PDA data extracted at 329 nm were compared to standards for the quantification of tabersonine, lochnericine, and hörhammericine (all in-house standards). Tryptophan (Sigma, St. Louis, MO, USA) and tryptamine (Sigma, St. Louis, MO, USA) were investigated using a previously described method and PDA data extracted at 218 nm (Peebles et al., 2005). Loganin (Fluka/Sigma, St. Louis, MO, USA) and secologanin (Fluka/Sigma, St. Louis, MO, USA) were measured using a previously describe method and PDA data extracted at 239 nm (Li et al., 2013).

### Promoter Analysis

*Catharanthus roseus* DNA sequences available in the Medicinal Plant Genomics Resource^2^ and NCBI^3^ databases were screened using BLASTN for sequences similar to the 16 (CAAAAGTATTATGATT) and 42 (CGCTATTTATCATATAATTATTTTACAATAATTAGTATTAGG) nucleotide CrBPF1 binding sites (van der Fits et al., 2000).

### Statistical Analyses

A two-tailed Student’s *t*-test was employed for statistical analyses. To identify statistically significant differences, results from β-estradiol induced hairy roots carrying the *CrBPF1* overexpression construct were compared with results from un-induced hairy roots carrying the *CrBPF1* overexpression construct. For qRT-PCR experiments, (^∗^) was used to represent *p* ≤ 0.05 and (^∗∗^) was used to represent *p* ≤ 0.01. For analyses of metabolite levels, (^∗^) was used to represent *p* ≤ 0.1 and (^∗∗^) was used to represent *p* ≤ 0.05.

## Results

### Generation of Transgenic Hairy Roots Expressing CrBPF1 under the Control of a β-Estradiol Inducible Promoter

CrBPF1 was identified by screening for proteins that bind the *STR* promoter. However, information regarding whether CrBPF1 plays a role in the regulation of other TIA or TIA-related genes is lacking, preventing determination of the role of CrBPF1 in regulation of these pathways. To address this deficiency, *C. roseus* transgenic hairy root lines that express *CrBPF1* under the control of an estradiol-inducible promoter (Zuo et al., 2000) were generated. Use of an estradiol-inducible promoter provides several advantages. An inducible promoter allows the timing and level of transgene expression to be controlled. This ability allows transgenic cultures to be grown without transgene expression, avoiding the possible deleterious consequences of long-term transgene expression. An inducible system also allows studies on the transient effects of transgene expression.

**FIGURE 2:**
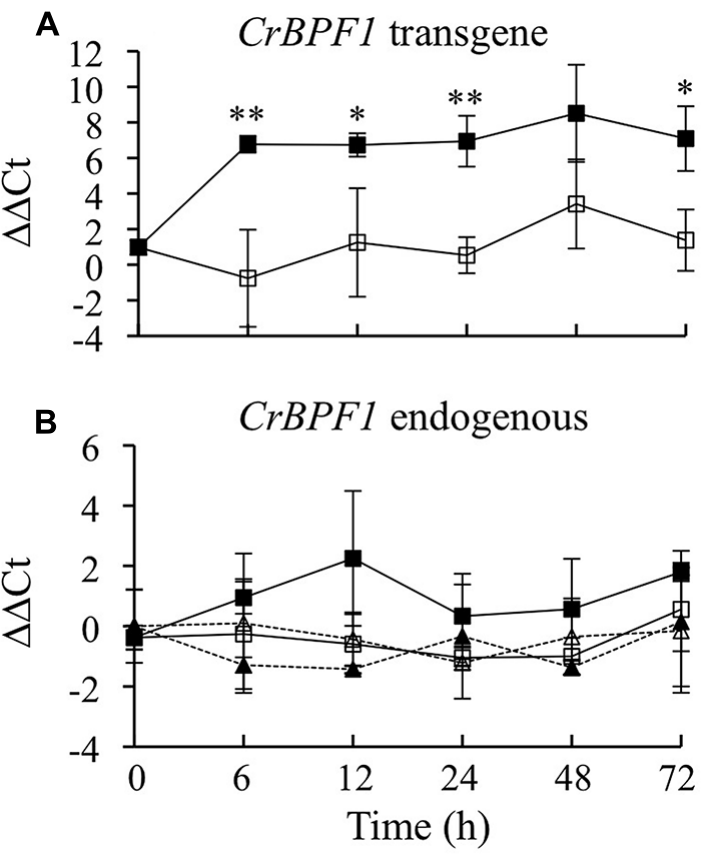
**Time course analysis of *CrBPF1* endogenous and transgene mRNA levels**. *CrBPF1* transcripts produced from the endogenous *CrBPF1* gene and the *CrBPF1* transgene were quantified independently by qRT-PCR using primers specific for each gene (Supplemental Table S1). As expected, the primer pair specific for transcripts produced by the *CrBPF1* transgene yields only a very low background signal for the control line (data not shown), which lacks the *CrBPF1* transgene. Results depicted are the following: un-induced CrBPF1-OE line (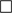, solid line), β-estradiol induced CrBPF1-OE line (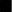, solid line), un-induced control line (Δ, dashed line) and β-estradiol induced control line (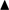, dashed line). **(A)** Relative *CrBPF1* transgene mRNA levels are indicated as ΔΔCt. Note that *CrBPF1* transgene mRNA levels were normalized versus *CrBPF1* endogenous gene mRNA levels in the un-induced control line at 0 h rather than against *CrBPF1* transgene mRNA levels in the un-induced control line at 0 h as the control line lacks the *CrBPF1* transgene. **(B)** Relative *CrBPF1* endogenous gene mRNA levels are indicated as ΔΔCt. A positive ΔΔCt value indicates that *CrBPF1* endogenous gene mRNA levels are higher in the indicated hairy root line grown for the indicated time under the indicated conditions than in the un-induced control line at 0 h. Negative ΔΔCt values indicate the reverse situation. Results are the average ΔΔCt of three biological replicates, with two technical replicates per biological replicate. Error bars indicate SD. *CrBPF1* mRNA levels in the un-induced versus induced CrBPF1-OE cultures differed at the same time point with: ^∗^*p* ≤ 0.05, ^∗∗^*p* ≤ 0.01 according to a Student’s *t*-test. The results of Student’s *t*-tests for the un-induced versus induced cultures of the control line are not depicted.

To generate transgenic hairy roots, *C. roseus* seedlings were inoculated with a mixture of *A. tumefaciens* cells transformed with either pERKT-CrBPF1 (Supplemental Figure S1) or pPZPROL (Hong et al., 2006). A total of 62 hairy root lines were screened for hygromycin resistance and seven lines were found to be resistant. These seven hairy root lines were confirmed to carry the *CrBPF1* transgene by using PCR to demonstrate the presence of a DNA fragment spanning part of the *CrBPF1* gene and an adjacent sequence from the pERKT-CrBPF1 vector. These positive lines were transferred to liquid culture. Lines with good adaption to growth in liquid culture were screened for β-estradiol inducible expression of *CrBPF1*. As expression of the *CrBPF1* transgene is particularly strongly induced by β-estradiol in the CrBPF1-OE line (**Figure 2**), and CrBPF1-OE also adapted well to growth in liquid media, further studies utilized this line.

To characterize β-estradiol inducible expression of *CrBPF1*, a time course experiment was performed using the CrBPF1-OE and control transgenic lines. The transgenic hairy root control line was generated previously (Li et al., 2013) by transforming *C. roseus* with pPZPROL alone, and thus lacks the β-estradiol-inducible *CrBPF1* transgene. The CrBPF1-OE and control lines were grown for 35 days and then transferred to fresh media with 0 μM (un-induced) or 20 μM (induced) β-estradiol. Tissue samples were collected 0, 6, 12, 24, 48, and 72 h after addition of β-estradiol. *CrBPF1* transcripts produced by the *CrBPF1* endogenous gene and by the *CrBPF1* transgene were quantified separately using qRT-PCR (**Figure 2**). *CrBPF1* transgene mRNA levels increased rapidly in the CrBPF1-OE line after addition of 20 μM β-estradiol, rising approximately 50 fold within 6 h, and remained high for at least 72 h. Un-induced cultures of the CrBPF1-OE line exhibited much lower transcript levels for the *CrBPF1* transgene than the induced cultures, indicating that β-estradiol is necessary for high-level expression of the *CrBPF1* transgene (**Figure 2A**). As expected, qRT-PCR reactions using a primer pair specific for the *CrBPF1* transgene produced only a very low signal from RNA isolated from the control line (data not shown), which lacks the *CrBPF1* transgene. Transcript levels for the *CrBPF1* endogenous gene were not significantly affected by treatment with β-estradiol and were similar in the CrBF1-OE and control lines (**Figure 2B**).

### Effects of *CrBPF1* Overexpression on the Indole and Terpenoid Pathways

CrBPF1 is one of only a few putative activators of the TIA pathway identified to date. Understanding the role played by CrBPF1 in regulation of the TIA pathway is important for developing strategies for *in vivo* manipulation of TIA production. Toward this end, the effects of *CrBPF1* overexpression on transcript levels of 31 genes were analyzed. The genes chosen for analysis include two genes from the indole pathway (*AS*α and *TDC*), six genes from the monoterpenoid pathway (*DXS1*, *DXS2A*, *DXS2B*, *G10H*, *CPR*, and *LAMT*) and ten genes from the TIA pathway (*STR*, *SGD*, *T16H1*, *T16H2*, *16OMT*, *D4H*, *DAT*, *PRX1*, *T19H*, and *MAT*). In addition, transcript levels of all of the cloned genes currently postulated to play a role in regulation of the TIA pathway were characterized. These genes include eight TIA transcriptional activators (*ORCA2*, *ORCA3*, *CrBPF1*, *CrMYC1*, *CrMYC2*, *CrWRKY1*, *CrWRKY2*, and *BIS1*) and five TIA transcriptional repressors (*ZCT1*, *ZCT2*, *ZCT3*, *GBF1*, and *GBF2*). In addition, the concentrations of 14 TIA metabolites were investigated, with nine of those metabolites being present at detectable levels. Both transcript and metabolite levels were tracked over a 72-h period in the CrBPF1-OE and control lines grown in the absence or presence of β-estradiol.

**FIGURE 3:**
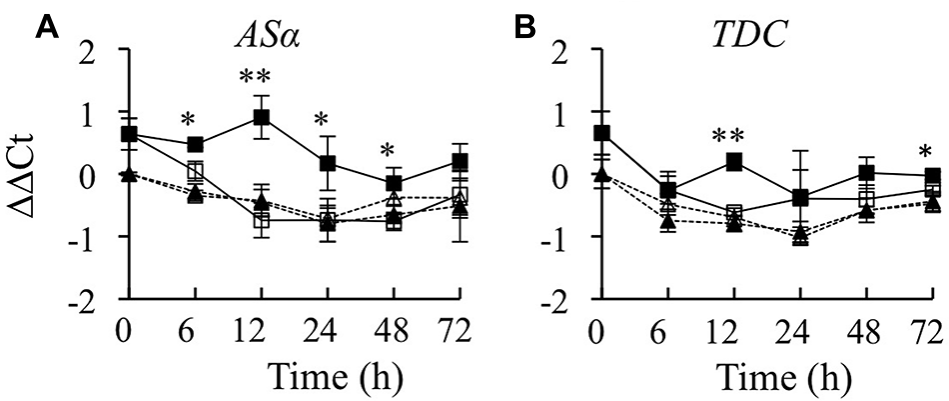
**Time course analysis of *AS*α and *TDC* mRNA levels**. Results depicted are the following: un-induced CrBPF1-OE line (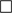, solid line), β-estradiol induced CrBPF1-OE line (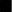, solid line), un-induced control line (Δ, dashed line) and β-estradiol induced control line (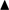, dashed line). Relative transcript levels are presented as ΔΔCt. Results shown are the following: **(A)** *AS*α mRNA levels and **(B)** *TDC* mRNA levels. Results are the average ΔΔCt values of three biological replicates with two technical replicates per biological replicate. Error bars indicate SD. Transcript levels in the un-induced versus induced CrBPF1-OE cultures differed at the same time point with: ^∗^*p* ≤ 0.05, ^∗∗^*p* ≤ 0.01 according to a Student’s *t*-test. The results of Student’s *t*-tests for the un-induced versus induced cultures of the control line are not depicted.

The production of TIAs is dependent on the synthesis of tryptamine in the indole pathway, the synthesis of secologanin in the terpenoid pathway and their subsequent coupling to form strictosidine. Thus, it was of interest to determine whether overexpression of *CrBPF1* affects expression of genes in the indole or terpenoid pathways. To characterize the effects of overexpressing *CrBPF1* on the indole pathway, *AS*α and *TDC* transcripts were quantified. *AS*α encodes the alpha subunit of anthranilate synthase, which catalyzes the first committed step in tryptophan synthesis. Overexpression of *CrBPF1* caused a significant increase in *AS*α transcript levels. *AS*α transcript levels were slightly higher in the induced versus un-induced CrBPF1-OE cultures 6 h after addition of β-estradiol, and reached a peak at the 12-h time point before beginning to decline. In contrast, addition of β-estradiol to the media had no significant effect on *AS*α transcript levels in the control line (**Figure 3A**). *TDC* encodes tryptophan decarboxylase, which catalyzes the formation of tryptamine from tryptophan. *TDC* transcript levels were higher in the induced versus un-induced CrBPF1-OE cultures 12 and 72 h after addition of β-estradiol. However, overexpression of *CrBPF1* caused only slight differences in *TDC* transcript levels, with a maximum difference of 75% higher transcript levels in the induced versus un-induced CrBPF1-OE cultures at the 12-h time point. Addition of β-estradiol to the media had no significant effect on *TDC* transcript levels in the control line (**Figure 3B**). An attempt was also made to analyze tryptophan and tryptamine levels in aliquots of the same tissue samples used to analyze gene expression. However, tryptophan and tryptamine levels were below the detection threshold in many of the samples.

**FIGURE 4:**
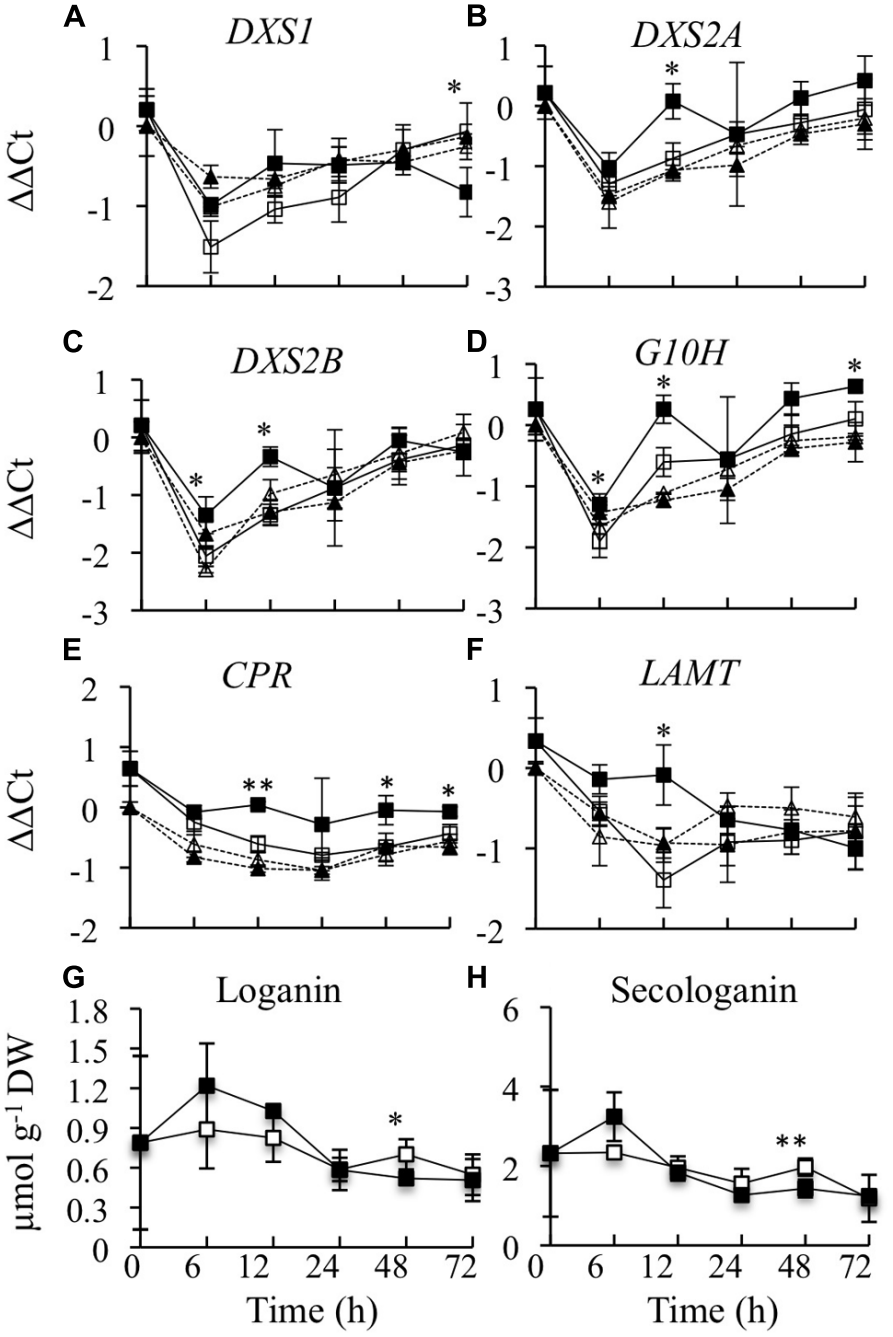
**Time course analyses of transcript and metabolite levels from the terpenoid pathway**. Results depicted are the following: un-induced CrBPF1-OE line (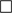, solid line), β-estradiol induced CrBPF1-OE line (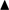, solid line), un-induced control line (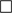, dashed line) and β-estradiol induced control line (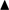, dashed line). Relative transcript levels are presented as ΔΔCt. Results shown are the following: **(A)** *DXS1* mRNA levels, **(B)** *DXS2A* mRNA levels, **(C)** *DXS2B* mRNA levels, **(D)** *G10H* mRNA levels **(E)** *CPR* mRNA levels, **(F)** *LAMT* mRNA levels, **(G)** loganin levels, and **(H)** secologanin levels. Results for transcript levels are the average ΔΔCt values of three biological replicates with two technical replicates per biological replicate. Results for metabolite levels are the averages of three biological replicates. Metabolite levels for the control line are not depicted. Error bars indicate SD. Transcript levels in the un-induced versus induced CrBPF1-OE cultures differed at the same time point with: ^∗^*p* ≤ 0.05, ^∗∗^*p* ≤ 0.01 according to a Student’s *t*-test. The results of Student’s *t*-tests for the un-induced versus induced cultures of the control line are not depicted. Metabolite levels in the un-induced versus induced CrBPF1-OE cultures differed at the same time point with: ^∗^*p* ≤ 0.1, ^∗∗^*p* ≤ 0.05 according to a Student’s *t*-test.

The expression levels of six genes in the terpenoid pathway were determined. Three of these genes (*DXS1*, *DXS2A*, and *DXS2B*) encode different isoforms of 1-deoxy-D-xylulose 5-phosphate synthase. *DXS2A* and *DXS2B* are induced by *ORCA3* overexpression, whereas *DXS1* is not regulated by ORCA3 (Han et al., 2013). Overexpression of *CrBPF1* had only a very modest effect on expression of the *DXS* genes. *DXS1* expression was 70% lower in the induced versus un-induced CrBPF1-OE cultures at the 72-h time point, but was not significantly altered at the other time points assayed. Addition of β-estradiol had a very slight effect on *DXS1* expression in the control line, causing a 6 to 30% increase in *DXS1* transcript levels at the 6, 12, and 72-h time points (**Figure 4A**). *DXS2A* expression was slightly induced by β-estradiol at the 12-h time point in the CrBPF1-OE line (**Figure 4B**). *DXS2B* transcript levels were increased 1.6 and 2.0 fold in the induced versus un-induced CrBPF1-OE cultures at the 6 and 12-h time points, respectively. However, *DXS2B* expression was also induced 1.5X in the induced versus un-induced control cultures at the 6-h time point, suggesting that application of β-estradiol, rather than increased *CrBPF1* expression, might be responsible for the alterations in *DXS2B* transcript levels (**Figure 4C**). *G10H* transcript levels were significantly higher in the induced versus un-induced CrBPF1-OE cultures at the 6, 12, and 72-h time points, but the differences in transcript levels were slight, reaching a maximum difference of less than twofold at the 12-h time point (**Figure 4D**). *CPR* transcript levels were significantly higher in the induced versus un-induced CrBPF1-OE cultures at the 12, 48, and 72-h time points, but the differences in transcript levels were slight, with a maximum difference of approximately 1.5 fold at the 48-h time point (**Figure 4E**). *LAMT* transcript levels were approximately 2.5 fold higher in the induced versus un-induced CrBPF1-OE cultures at the 12-h time point, but were not significantly different at the other time points analyzed (**Figure 4F**). Addition of β-estradiol had no significant effects on transcript levels of *G10H*, *CPR*, or *LAMT* in the control line. The levels of loganin and secologanin were also determined in aliquots of the same tissue samples used for gene expression analyses. The addition of 20-μM β-estradiol to the media caused no substantial alterations in the levels of either of these metabolites over the time period analyzed (**Figures 4G,H**).

### Effects of *CrBPF1* Overexpression on TIA Biosynthetic Gene mRNA Levels

**FIGURE 5:**
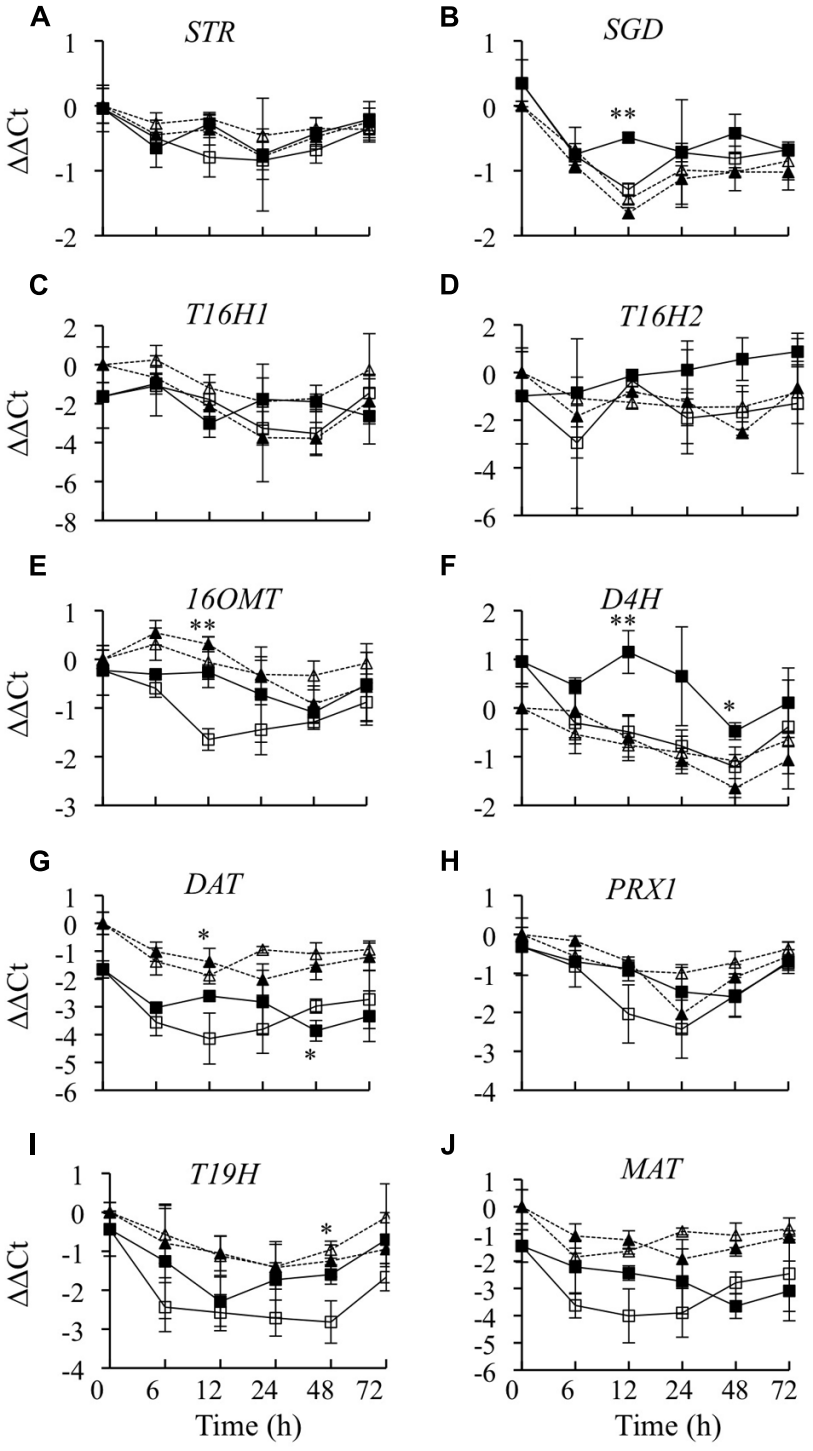
**Time course analysis of TIA biosynthetic gene mRNA levels**. Results depicted are the following: un-induced CrBPF1-OE line (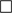, solid line), β-estradiol induced CrBPF1-OE line (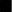, solid line), un-induced control line (Δ, dashed line) and β-estradiol induced control line (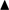, dashed line). Relative transcript levels are presented as ??Ct. Results shown are the following: **(A)** *STR* mRNA levels, **(B)** *SGD* mRNA levels **(C)** *T16H1* mRNA levels, **(D)** *T16H2* mRNA levels, **(E)** *16OMT* mRNA levels, **(F)** *D4H* mRNA levels, **(G)** *DAT* mRNA levels, **(H)** *PRX1* mRNA levels, **(I)** *T19H* mRNA levels and **(J)** *MAT* mRNA levels. Results are the average ΔΔCt values of three biological replicates with two technical replicates per biological replicate. Error bars indicate SD. Transcript levels in the un-induced versus induced CrBPF1-OE cultures differed at the same time point with: ^∗^*p* ≤ 0.05, ^∗∗^*p* ≤ 0.01 according to a Student’s *t*-test. The results of Student’s *t*-tests for the un-induced versus induced cultures of the control line are not depicted.

To characterize the effects of overexpressing *CrBPF1* on the TIA pathway, transcript levels of ten TIA biosynthetic genes were characterized. *STR* and *SGD* encode the enzymes that catalyze the first two steps in TIA biosynthesis. Overexpression of *CrBPF1* had no significant effects on *STR* transcript levels (**Figure 5A**). *SGD* transcript levels were 75% higher in the induced versus un-induced CrBPF1-OE cultures at the 12-h time point, but were not significantly altered by *CrBPF1* overexpression at the other time points assayed (**Figure 5B**). *T16H*, *16OMT*, *D4H*, and *DAT* encode enzymes that catalyze different steps in the pathway leading from tabersonine to formation of vindoline. Overexpression of *CrBPF1* had no significant effects on *T16H1* (**Figure 5C**) or *T16H2* (**Figure 5D**) transcript levels. *16OMT* transcript levels were 2.6-fold higher in induced versus un-induced CrBPF1-OE cultures at the 12-h time point, but were not significantly altered by *CrBPF1* overexpression at the other time points assayed (**Figure 5E**). *D4H* transcript levels were significantly higher in the induced versus un-induced CrBPF1-OE cultures at the 12 and 48-h time points, but not at the other time points analyzed (**Figure 5F**). The effects of *CrBPF1* overexpression on *DAT* transcript levels were more complex. *DAT* transcript levels were approximately 3-fold higher in the induced versus un-induced CrBPF1-OE cultures at the 12-h time point, but were almost twofold lower in the induced versus un-induced CrBPF1-OE cultures at the 48-h time point. Interestingly, *DAT* transcript levels in the CrBPF1-OE line were consistently below those in the control line (**Figure 5G**). *PRX1* encodes a vacuolar class III peroxidase that catalyzes the synthesis of α-3′, 4′-anhydrovinblastine from catharanthine and vindoline. Overexpression of *CrBPF1* had no significant effects on *PRX1* transcript levels (**Figure 5H**). *T19H* transcript levels were 2.3-fold higher in induced versus un-induced CrBPF1-OE cultures at the 48-h time point, but were not significantly altered by CrBPF1 overexpression at the other time points assayed (**Figure 5I**). Overexpression of CrBPF1 had no significant effects on *MAT* transcript levels (**Figure 5J**). Addition of β-estradiol to the media had little effect on TIA biosynthetic gene expression in the control line. Where TIA biosynthetic gene transcript levels did vary between control cultures grown in the presence of 0 versus 20 μM β-estradiol, transcript levels were typically higher in the cultures grown on 0 μM β-estradiol. For example, *T16H1* transcript levels at the 48-h time point and *DAT* and *PRX1* transcript levels at the 24-h time point were higher in control cultures grown on 0 μM β-estradiol than on 20 μM β-estradiol (**Figure 5**). These results are in contrast to the results observed for the CrBPF1-OE cultures, where addition of 20-μM β-estradiol to the media tended to cause increased transcript levels.

### Effects of *CrBPF1* Overexpression on TIA Metabolite Levels

**FIGURE 6:**
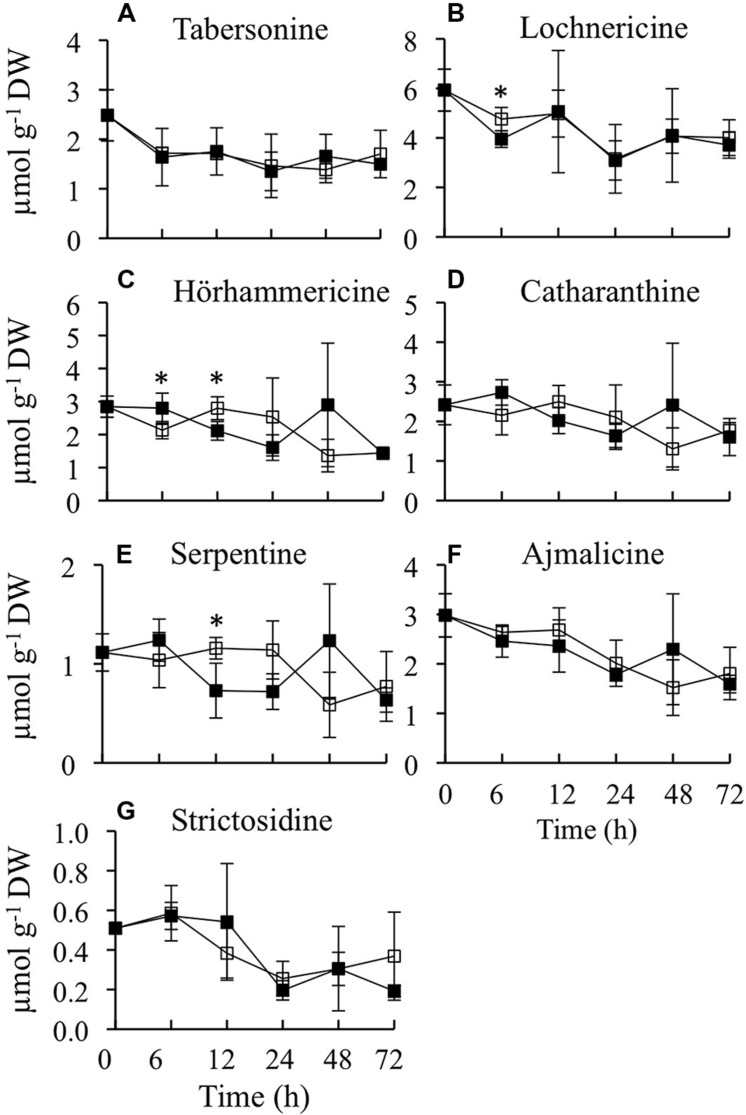
**Time course analysis of TIA metabolite levels**. Results depicted are the following: un-induced CrBPF1-OE line (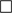, solid line) and β-estradiol induced CrBPF1-OE line (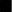, solid line). Metabolite levels shown are the following: **(A)** tabersonine levels, **(B)** lochnericine levels **(C)** hörhammericine levels, **(D)** catharanthine levels, **(E)** serpentine levels, **(F)** ajmalicine levels and **(G)** strictosidine levels. Results are the average values of three biological replicates. Error bars indicate SD. Metabolite levels in the un-induced versus induced CrBPF1-OE cultures differed at the same time point with: ^∗^*p* ≤ 0.1 according to a Student’s *t*-test.

As *CrBPF1* overexpression affects the transcript levels of many of the genes involved in synthesis of TIAs or TIA precursors, it was of interest to determine whether overexpression of *CrBPF1* affects TIA metabolite levels. Toward that end, the levels of ten TIA metabolites were analyzed over a 72-h period in the CrBPF1-OE line grown in the presence or absence of 20-μM β-estradiol, with seven of those metabolites being present at detectable levels (**Figure 6**). The metabolites analyzed were tabersonine, lochnericine, hörhammericine, catharanthine, serpentine, ajmalicine, strictosidine, vindoline, vincristine, and vinblastine, with the levels of the last three being below the detection threshold. Overexpression of *CrBPF1* had only modest effects on the levels of the other seven metabolites, with the largest statistically significant effect being ∼40% lower serpentine levels in the induced versus un-induced CrBPF1-OE cultures at the 12-h time point. The levels of the same metabolites were also analyzed in the control line, at 0 and 24 h after transfer to fresh media with 0 or 20 μM β-estradiol. Addition of 20-μM β-estradiol to the media had little effect on the levels of any of the TIA metabolites analyzed in the control line (data not shown).

### Effects of *CrBPF1* Overexpression on TIA Regulatory Genes

**FIGURE 7:**
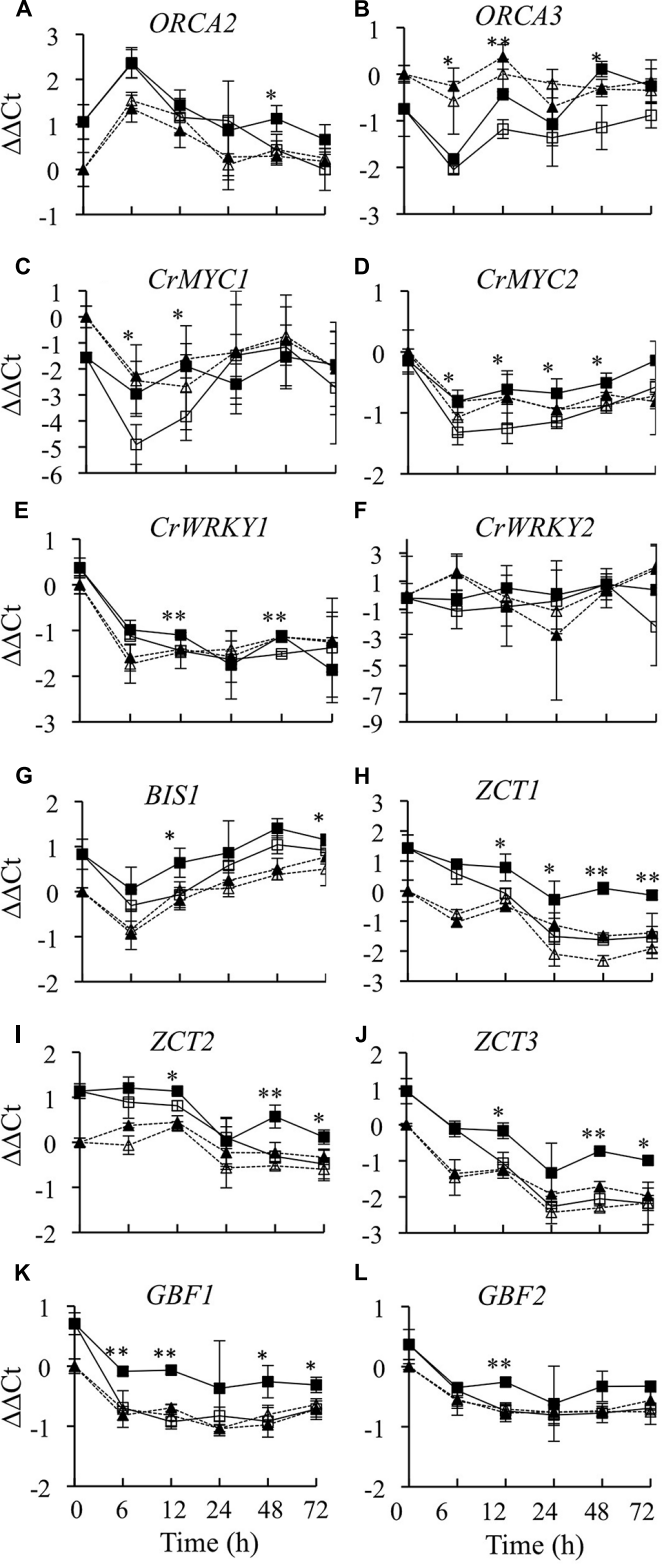
**Time course analysis of TIA regulatory gene mRNA levels**. Results depicted are the following: un-induced CrBPF1-OE line (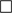, solid line), β-estradiol induced CrBPF1-OE line (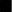, solid line), un-induced control line (Δ, dashed line) and β-estradiol induced control line (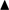, dashed line). Relative transcript levels are presented as ΔΔCt. Results shown are the following: **(A)** *ORCA2* mRNA levels, **(B)** *ORCA3* mRNA levels **(C)** *CRMYC1* mRNA levels, **(D)** *CrMYC2* mRNA levels, **(E)** *CrWRKY1* mRNA levels, **(F)** *CrWRKY2* mRNA levels, **(G)** *BIS1* mRNA levels, **(H)** *ZCT1* mRNA levels, **(I)** *ZCT2* mRNA levels, **(J)** *ZCT3* mRNA levels, **(K)** *GBF1* mRNA levels and **(L)** *GBF2* mRNA levels. Results are the average ΔΔCt values of three biological replicates with two technical replicates per biological replicate. Error bars indicate SD. Transcript levels in the un-induced versus induced CrBPF1-OE cultures differed at the same time point with: ^∗^*p* ≤ 0.05, ^∗∗^*p* ≤ 0.01 according to a Student’s *t*-test. The results of Student’s *t*-tests for the un-induced versus induced cultures of the control line are not depicted.

In addition to regulating expression of biosynthetic genes directly, a transcriptional regulator may affect expression of biosynthetic genes by altering the activities of other transcriptional regulators. To determine whether overexpression of *CrBPF1* affects the activities of TIA transcriptional regulators, transcript levels for all the postulated TIA regulators for which genes have been cloned were analyzed in CrBPF1-OE and control cultures grown on 0 or 20 μM β-estradiol. Currently, eight transcriptional activators (*ORCA2*, *ORCA3*, *CrBPF1*, *CrMYC1*, *CrMYC2*, *CrWRKY1*, *CrWRKY2*, and *BIS1*) and five transcriptional repressors (*ZCT1*, *ZCT2*, *ZCT3*, *GBF1*, and *GBF2*) are postulated to function in regulation of the TIA pathway. Overexpression of *CrBPF1* had little effect on *ORCA2* transcript levels, with the only statistically significant effect being an approximately 60% increase in *ORCA2* transcript levels in the induced versus un-induced CrBPF1-OE cultures at the 48-h time point (**Figure 7A**). Overexpression of *CrBPF1* had a more consistent effect on *ORCA3* expression, with *ORCA3* transcript levels being significantly higher in the induced versus un-induced CrBPF1-OE cultures at the 6, 12, and 48-h time points, reaching a maximum difference of 2.4 fold at the 48-h time point (**Figure 7B**). To determine whether *CrBPF1* affects its own expression, transcripts produced by the endogenous *CrBPF1* gene were analyzed in the CrBPF1-OE and control lines grown on 0 or 20 μM β-estradiol. Increased expression of the *CrBPF1* transgene had no significant effect on *CrBPF1* endogenous gene transcript levels, indicating that CrBPF1 does not regulate its own expression at the steady-state transcriptional level (**Figure 2B**). *CrMYC1* transcript levels were almost fourfold higher in induced versus un-induced CrBPF1-OE cultures at the 6 and 12-h time points, but were not significantly altered at later time points (**Figure 7C**). Overexpression of *CrBPF1* had smaller, but more consistent effects on *CrMYC2* transcript levels, with 30–60% higher *CrMYC2* transcript levels in the induced versus un-induced CrBPF1-OE cultures at all time points assayed, except for the latest time point (**Figure 7D**). Overexpression of *CrBPF1* also had a modest effect on *CrWRKY1* transcript levels, which exhibited statistically significant 30% increases in the induced versus un-induced CrBPF1-OE cultures at the 12 and 48-h time points (**Figure 7E**). In contrast, overexpression of *CrBPF1* had no statistically significant effects on *CrWRKY2* transcript levels (**Figure 7F**). Overexpression of CrBPF1 caused a statistically significant 60% increase in *BIS1* transcript levels at the 12-h time point and a 20% increase at the 72-h time point (**Figure 7G**).

Overexpression of *CrBPF1* had a comparatively large effect on expression of the *ZCT1* transcriptional repressor. *ZCT1* transcript levels were twofold to threefold higher in the induced versus un-induced CrBPF1-OE cultures at all time points assayed, except for the earliest, 6-h, time point (**Figure 7H**). Overexpression of *CrBPF1* also caused significant increases in *ZCT2* (**Figure 7I**) and *ZCT3* (**Figure 7J**) expression levels at the 12, 48, and 72-h time points. Overexpression of *CrBPF1* caused a larger increase in *ZCT3* than in *ZCT2* transcript levels, with *ZCT3* transcript levels being approximately 2–2.5 fold higher in the induced versus un-induced CrBPF1-OE cultures, as opposed to approximately 1.25–2 fold increases in *ZCT2* transcript levels. Overexpression of *CrBPF1* had modest, but fairly consistent, effects on *GBF1* expression. *GBF1* transcript levels were significantly higher in the induced versus un-induced CrBPF1-OE cultures at all time points assayed, except for the 24-h time point, where the mean *GBF1* transcript levels were higher in the induced than in the un-induced CrBPF1-OE cultures, but the difference was not statistically significant (**Figure 7K**). Overexpression of *CrBPF1* had only a very slight effect on *GBF2* transcript levels, with the only statistically significant difference being an approximately 40% increase in *GBF2* transcript levels in the induced versus the un-induced CrBPF1-OE cultures at the 12-h time point (**Figure 7L**). Addition of 20-μM β-estradiol to the media had little effect on expression of TIA regulatory genes in the control cultures.

### TIA Promoter Analysis

DNase1 fingerprinting resulted in the identification of 16 and 42 nt CrBPF1 binding sites within the BA fragment of the *C. roseus STR* promoter (van der Fits et al., 2000). *C. roseus* DNA sequences available in the Medicinal Plant Genomics Resource^2^ and NCBI^3^ databases were searched using BLASTN for sequences similar to these 16 and 42-nucleotide CrBPF1 binding sites. Examination of the top 50 matches for the 16 and 42-nt sequences from the Medicinal Plant Genomics Resource did not reveal any matches to promoter sequences from genes believed to be involved in TIA biosynthesis, with the exception of *STR*. In contrast, searches of the sequences available in GenBank revealed several partial matches to the 5′ regions of genes involved in TIA biosynthesis. The best matches to sequences lying within approximately 1,500 bp 5′ of a transcription start site are listed in Supplemental Table S3. In addition to *STR*, partial matches for both the 16 and 42-nt sequences were found for *ORCA3* and *TDC*. However, the spacing between these partial matches was much larger for *ORCA3* and *TDC* than for *STR*. The 5′ ends of the 16 and 42 nt sequences are 70 bp apart in the *STR* promoter, but are 365 and 552 bp apart in the *ORCA3* and *TDC* promoters, respectively. Partial matches to the 16 nt sequence, but not to the 42 nt sequence, were found for *CPR*, *PRX1*, *BIS1*, and *CrWRKY1*. A partial match to the 42 nt sequence, but not to the 16 nt sequence, was found for *DXS2B*.

## Discussion

CrBPF1 was identified as a MYB-like protein that binds the BA region of the *C. roseus STR* promoter (van der Fits et al., 2000). Although thirteen transcriptional regulators have been postulated to act in regulation of the TIA pathway, the effects of most of these transcriptional regulators on expression of the majority of the known TIA biosynthetic and regulatory genes have not yet been determined. To address this deficiency, a *C. roseus* transgenic hairy root line that expresses *CrBPF1* under the control of a β-estradiol inducible promoter was generated and characterized. Addition of β-estradiol to the medium causes a large and rapid induction of *CrBPF1* transgene expression in the CrBPF1-OE line but does not affect *CrBPF1* expression in a control line, indicating that the presence of the transgene is necessary for increased *CrBPF1* transcript levels.

Terpenoid indole alkaloid production is dependent on the synthesis of precursors by both the indole and terpenoid pathways, the combination of these precursors by STR and subsequent reactions carried out by different branches of the TIA pathway. Overexpression of *CrBPF1* caused increased expression of the majority of the genes analyzed from these pathways. However, these effects were typically transient and of limited magnitude. Interestingly, *CrBPF1* overexpression may cause decreases in *DAT* expression. Although overexpression of *CrBPF1* caused increased *DAT* transcript levels after 12 h, *DAT* transcript levels decreased after 48 h. In addition, *DAT* transcript levels were consistently lower in the CrBPF1-OE line than in the control line over the entire time course, suggesting that increased *CrBPF1* expression tends to have a negative effect on *DAT* activity. Interestingly, *DAT* expression has been shown to be strongly decreased in response to overexpression of *ORCA2* (Li et al., 2013). The finding that *CrBPF1* overexpression does not have a significant effect on *STR* transcript levels might appear somewhat unexpected, given that CrBPF1 is known to bind the *STR* promoter (van der Fits et al., 2000). However, this finding is consistent with previous work suggesting that CrBPF1 might have only a limited effect on *STR* expression (van der Fits et al., 2000). Binding of CrBPF1 to the *STR* promoter is only part of the process promoting *STR* transcription, with binding of additional factors to other *cis* sequences playing an important role in regulation of *STR* activity (van der Fits et al., 2000; Chatel et al., 2003). Consequently, overexpression of *CrBPF1* alone is insufficient to cause significant alterations in *STR* transcript levels.

To determine whether overexpression of *CrBPF1* affects the expression of other regulators, transcript levels for all genes postulated to encode TIA regulators were analyzed. Overexpression of *CrBPF1* caused increased expression of all of the other TIA transcriptional activators except *CrWRKY2*. In addition, expression of the *CrBPF1* transgene did not affect expression of the *CrBPF1* endogenous gene, indicating that *CrBPF1* does not regulate its own expression. Interestingly, overexpression of *CrBPF1* also caused increased transcript levels for all five of the genes postulated to encode TIA transcriptional repressors. These results suggest that *CrBPF1* overexpression could be altering the transcript levels of some of the biosynthetic genes characterized indirectly, by altering the activities of other TIA regulatory genes. The results of a promoter analysis are consistent with this possibility. Partial matches for both the 16 and 42-nt CrBPF1 binding sites from the *STR* promoter were found in the promoters of only *ORCA3* and *TDC*, among the genes analyzed as part of this study. However, the spacing between these partial matches was much greater for *ORCA3* and *TDC* than for *STR*. Partial matches to the 16-nt CrBPF1 binding sequence were also found for the *CPR*, *PRX1*, *BIS1*, and *CrWRKY1* promoters and to the 42-nt sequence for *DXS2B*.

As *CrBPF1* overexpression affects the activities of several TIA biosynthetic genes and of most of the TIA regulatory genes analyzed, it was of interest to determine whether *CrBPF1* overexpression also affects TIA metabolite levels. Toward that end the levels of 14 metabolites from different parts of the TIA and feeder pathways were analyzed, with the levels of nine of those metabolites being above the detection threshold. The results of these analyses indicate that *CrBPF1* overexpression causes only slight, and transient, alterations in lochnericine, hörhammericine and serpentine levels, typically causing decreased levels of these metabolites. Lochnericine is the precursor for synthesis of hörhammericine, suggesting that flux to this branch of the TIA pathway may be altered by *CrBPF1* overexpression. Serpentine is synthesized via a different branch of the TIA pathway. The relatively minor effects of *CrBPF1* overexpression on TIA metabolite levels may be due to the fact that, although *CrBPF1* overexpression increases the activities of many TIA and related genes, these increases in gene expression are typically of limited magnitude and duration.

The results of this work indicate that although *CrBPF1* regulates a high percentage of the genes analyzed, the effects of *CrBPF1* overexpression on gene activity levels tend to be of comparatively limited magnitude and are often transient. These modest alterations in TIA biosynthetic gene expression may help explain the limited effects of *CrBPF1* overexpression on the levels of the TIA metabolites analyzed. The relatively minor increases in gene expression may be due to the fact that *CrBPF1* overexpression causes increased expression of all five TIA transcriptional repressors, in addition to causing increased activity of most of the TIA transcriptional activators. In contrast, overexpression of *ORCA2* (Li et al., 2013) and *ORCA3* (Peebles et al., 2009) causes increased expression of the *ZCT* transcriptional repressors, but not of the *GBF* transcriptional repressors. As overexpression of *CrBPF1* has comparatively large effects on expression of TIA transcriptional repressors, future characterization of these repressors is expected to yield further insight into the mechanism by which CrBPF1 helps regulate the TIA and related pathways. The simultaneous activation of transcriptional repressors and activators has been proposed to act as part of a “fine tune” mechanism for regulating TIA production (Memelink and Gantet, 2007). The findings reported here support this model and suggest that CrBPF1 plays a role in the “fine tune” regulation of TIA metabolism.

## Conflict of Interest Statement

The authors declare that the research was conducted in the absence of any commercial or financial relationships that could be construed as a potential conflict of interest.

## Footnotes

1 www.neseed.com

2 http://medicinalplantgenomics.msu.edu/mpgr_blast.shtml#aln2

3 https://blast.ncbi.nlm.nih.gov/Blast.cgi?PAGE_TYPE=BlastSearch
