# Supplementary material for: CrBPF1 overexpression alters transcript levels of terpenoid indole alkaloid biosynthetic and regulatory genes
Source: Front Plant Sci. 2015 Oct 1;6:818. doi: 10.3389/fpls.2015.00818 (PMC4589645; doi:10.3389/fpls.2015.00818)
Supplement: Supplementary file 1 [file Table_1.DOCX]

**Supplemental Table 1. Primers and probes used for qRT-PCR analyses.** This table describes the nucleotides sequences of the primer pairs used during qRT-PCR analyses. The numbers of the probes from the Roche Universal Probe Library used to detect the amplicons produced during these assays are also indicated. Note that qRT-PCR analyses of transcripts from the *CrMYC1*, *CrMYC2*, *CrWRKY1*, *CrWRKY2*, *CrBPF1* endogenous (CrBPF1en), *CrBPF1* trans (CrBPF1tr), *BIS1*, *T16H2*, *MAT*, *T19H*, *DXS1* and *DXS2B* genes were performed using SYBR Green and therefore did not use a probe from the Roche Universal Probe Library. The CrBPF1tr_F primer binds to a region of the transcript encoded by the pERKT-CrBPF1 plant transformation construct that is coded by sequences present on the vector, rather than on the *CrBPF1* gene.

| **Primer name** | **Primer sequence** | **Probe #** |
| --- | --- | --- |
| ORCA2_F | tcaacaacgattttgatttttca | 126 |
| ORCA2_R | tccgaagcataatttggtga |  |
| ORCA3_F | ttccagctcggaattgactt |  |
| ORCA3_R | cgaccaatttagaaaatctgcaa |  |
| CrBPF1en_F | tgcgtgcttttgacaatgc |  |
| CrBPF1en_R | aaactccaaactcagaaattaatccg |  |
| CrBPF1tr_F | tttggagaggacacgctgaa |  |
| CrBPF1tr_R | cgctactgcagccagcaat |  |
| CrMYC1_F | gtttccgatgaacagcgctac |  |
| CrMYC1_R | cctcattcatggcattggc |  |
| CrMYC2_F | aaaaacaaccaccctgcagc |  |
| CrMYC2_R | aatcggctccccattctca |  |
| CrWRKY1_F | ttggtcccgacgatattcgt |  |
| CrWRKY1_R | tcgtttgtaagagcctttccg |  |
| CrWRKY2_F | tgctagaaataacggccagaat |  |
| CrWRKY2_R | ccctctttcaactgtagaaaac |  |
| BIS1_F | agagccgctcgtactcccat |  |
| BIS1_R | ccttgggaggtcaaattctca |  |
| ZCT1_F | tctcggaggtcatatgagacg | 58 |
| ZCT1_R | cgcctttgcaacaggtttat |  |
| ZCT2_F | aaaaacccttaattttctccatatttc | 9 |
| ZCT2_R | tctcgtacgcttcatcggta |  |
| ZCT3_F | cgcagcaacacaatattcctt | 143 |
| ZCT3_R | acacttgtagagaagcttaggagga |  |
| GBF1_F | cagagaaagctatgagggcaag | 18 |
| GBF1_R | cacccatcaccttttcagttg |  |
| GBF2_F | agaatctgctcggcgatcta | 96 |
| GBF2_R | cgctgagccaattcatca |  |
| TDC_F | aaaatgttcgaagaatgggttaga | 109 |
| TDC_R | gtttctcggtaccacaatttcg |  |
| DXS1_F | cttcccaagacggtgctttg |  |
| DXS1_R | ggtcataagccctttgcatga |  |
| DXS2A_F | gagcactcagcagtgcctta | 125 |
| DXS2A_R | gcttcccgtaattgcctaaa |  |
| DXS2B_F | atagagcagggctggtgggt |  |
| DXS2B_R | ggcaaccatgtgcatcaatt |  |
| G10H_F | gtacaggaactaattgcgtattgc | 106 |
| G10H_R | cgacgtcaaccgcttctc |  |
| CPR_F | ttgcagtgaggaaggagctt | 67 |
| CPR_R | aatccaaatgggtgcaagaa |  |
| STR_F | ttctatggctttttgaaggttaca | 63 |
| STR_R | catatatgtagcagcagacactcaaa |  |
| D4H_F | tgaactttcatgctgctacactc | 143 |
| D4H_R | ccagcctttgtctcatcaaaa |  |
| DAT_F | cacggtagcagggaaatcag | 142 |
| DAT_R | ctggaaatggcaaagattgg |  |
| EF1_F | ccgtctcccacttcaggat | 119 |
| EF1_R | cacgaccaacagggacagta |  |
| UBQ11_F | cgtcaaggctaaaattcagga | 31 |
| UBQ11_R | gaatattgtagtcggccaaggt |  |
| SGD_F | cattggtgaaccgtgctatg | 121 |
| SGD_R | agattgtagagtccagatggaaca |  |
| T16H1_F | tgattttaaaggaaattcattcgag | 77 |
| T16H1_R | tgcccggacatatccttc |  |
| T16H2_F | agtcaatgcatgggctatcg |  |
| T16H2_R | caccaccaaacggcagatac |  |
| LAMT_F | gaagcccacccaatgaaa | 9 |
| LAMT_R | ggtagcaagaattttgggagtaac |  |
| 16OMT_F | tggagagtataagttcttgtttgagg | 139 |
| 16OMT_R | cggtaccgcctcctatatca |  |
| PRX1_F | actttgcaacaagggcagac | 31 |
| PRX1_R | gggcacttgtgttgcttgt |  |
| AS alpha_F | aggctaaggagcacatcctg | 151 |
| AS alpha_R | cgttcaaaacgctgacttagg |  |
| T19H_F | gaggcactccggttacatcc |  |
| T19H_R | tctctcgctattgcccaagc |  |
| MAT_F | cgaaggccattgagtttggt |  |
| MAT_R | gaaaggcaaagattggcca |  |
