# Supplementary material for: CrBPF1 overexpression alters transcript levels of terpenoid indole alkaloid biosynthetic and regulatory genes
Source: Front Plant Sci. 2015 Oct 1;6:818. doi: 10.3389/fpls.2015.00818 (PMC4589645; doi:10.3389/fpls.2015.00818)
Supplement: Supplementary file 2 [file Table_2.DOCX]

**Supplemental Table 2. Comparison of *EF1* and *UBQ11* ∆CT values**. CT values for the indicated gene, hairy root line, time point and treatment condition were subtracted from the mean CT value for the same gene in the control line at 0 h. Results are the average ∆CT values of three biological replicates with two technical replicates per biological replicate.

|  |  |  | *EF1* | | *UBQ11* | |
| --- | --- | --- | --- | --- | --- | --- |
| Line | Time point | Treatment | Mean | STD DEV | Mean | STD DEV |
| Control | 0 |  | 0.00 | 0.20 | 0.00 | 0.06 |
| Control | 6 | Un-induced | 0.67 | 0.32 | 0.12 | 0.55 |
| Control | 12 | Un-induced | 0.87 | 0.11 | 0.34 | 0.07 |
| Control | 24 | Un-induced | 0.93 | 0.07 | 0.25 | 0.09 |
| Control | 48 | Un-induced | 0.63 | 0.05 | 0.30 | 0.03 |
| Control | 72 | Un-induced | 0.22 | 0.36 | 0.09 | 0.51 |
| Control | 0 |  | 0.00 | 0.20 | 0.00 | 0.06 |
| Control | 6 | Induced | 0.93 | 0.21 | 0.21 | 0.10 |
| Control | 12 | Induced | 1.05 | 0.03 | 0.65 | 0.24 |
| Control | 24 | Induced | 0.64 | 0.56 | -0.06 | 0.63 |
| Control | 48 | Induced | 0.73 | 0.14 | 0.36 | 0.17 |
| Control | 72 | Induced | 0.04 | 1.01 | -0.33 | 0.89 |
| CrBPF1-OE | 0 |  | -0.41 | 0.23 | 0.38 | 0.29 |
| CrBPF1-OE | 6 | Un-induced | 1.08 | 0.19 | 0.71 | 0.20 |
| CrBPF1-OE | 12 | Un-induced | 1.43 | 0.22 | 0.99 | 0.33 |
| CrBPF1-OE | 24 | Un-induced | 1.11 | 0.23 | 0.67 | 0.04 |
| CrBPF1-OE | 48 | Un-induced | 1.16 | 0.26 | 0.67 | 0.25 |
| CrBPF1-OE | 72 | Un-induced | 0.29 | 0.42 | -0.02 | 0.56 |
| CrBPF1-OE | 0 |  | -0.41 | 0.23 | 0.38 | 0.29 |
| CrBPF1-OE | 6 | Induced | 0.80 | 0.22 | 1.23 | 0.28 |
| CrBPF1-OE | 12 | Induced | 0.50 | 0.20 | 1.05 | 0.16 |
| CrBPF1-OE | 24 | Induced | 0.16 | 0.53 | 0.41 | 0.72 |
| CrBPF1-OE | 48 | Induced | 0.61 | 0.10 | 0.61 | 0.14 |
| CrBPF1-OE | 72 | Induced | 0.07 | 0.75 | 0.13 | 0.67 |
