# Supplementary material for: CrBPF1 overexpression alters transcript levels of terpenoid indole alkaloid biosynthetic and regulatory genes
Source: Front Plant Sci. 2015 Oct 1;6:818. doi: 10.3389/fpls.2015.00818 (PMC4589645; doi:10.3389/fpls.2015.00818)
Supplement: Supplementary file 3 [file Table_3.DOCX]

**Supplemental Table 3. Potential matches to CrBPF1 binding sites.** DNase 1 footprinting was used previously to identify 16 and 42 nt CrBPF1 binding sites within the BA fragment of the *STR* promoter (van der Fits et al., 2000). These 16 and 42 nt sequences were used to search *C. roseus* sequences in GenBank and the Medicinal Plant Genomics Resource. The most significant matches to potential promoter regions from the GenBank search are shown below. Query sequence; 16 or 42 nt CrBPF1 binding site; Query nucleotide match, nucleotides from 16 or 42 nt CrBPF1 binding site that matched indicated gene; Gene, gene with match in possible promoter region to indicated query sequence; GenBank ID, GenBank ID for indicated gene; Subject nucleotide match, nucleotides from indicated gene sequence that matched 16 or 42 nt CrBPF1 binding site; Match, number of nucleotides within indicated sequences that were identical; Score, GenBank score for indicated match; Expect, GenBank expect value for indicated match; CrBPF1 regulated, indicated gene affected by CrBPF1 overexpression; nt, not tested.

| Query sequence | Query nucleotide match | Gene | GenBank ID | Subject nucleotide match | Match | Score | Expect | CrBPF1 regulated |
| --- | --- | --- | --- | --- | --- | --- | --- | --- |
| 42 nt | 26-37 | 7-Deoxyloganic acid hydroxylase | KP963957.1 | 326-315 | 12 of 12 | 24.3 | 1.5 | nt |
| 16 nt | 5-14 | Alternative oxidase | AB055060.1 | 1741-1750 | 10 of 10 | 20.3 | 4 | nt |
| 16 nt | 4-12 | CPR | Y09417.1 | 319-327 | 9 of 9 | 18.3 | 16 | yes |
| 42 nt | 6-21 | CYC07 | D26058.1 | 1506-1491 | 15 of 16 | 24.3 | 1.5 | nt |
| 16 nt | 8-16 | Desacetoxyvindoline-4-hydroxylase-like protein | GU363550.1 | 733-741 | 9 of 9 | 18.3 | 16 | nt |
| 42 nt | 12-25 | DXS2B | KC625535.1 | 42-55 | 14 of 14 | 28.2 | 0.097 | yes* |
| 42 nt | 14-25 | GES | KP963953.1 | 251-262 | 12 of 12 | 24.3 | 1.5 | nt |
| 16 nt | 2-14 | GES | KP963953.1 | 1127-1115 | 12 of 13 | 18.3 | 16 | nt |
| 16 nt | 4-12 | GES | KP963953.1 | 561-553 | 9 of 9 | 18.3 | 16 | nt |
| 42 nt | 15-25 | Hydroxymethylbutenyl 4-diphosphate synthase | JN217103.1 | 70-60 | 11 of 11 | 22.3 | 6 | nt |
| 16 nt | 8-16 | BIS1 | KP963956.1 | 308-316 | 9 of 9 | 18.3 | 16 | yes |
| 16 nt | 2-10 | Isochorismate synthase | AY555149.1 | 649-641 | 9 of 9 | 18.3 | 16 | nt |
| 16 nt | 2-10 | ORCA3 | AJ251250.1 | 322-314 | 9 of 9 | 18.3 | 16 | yes |
| 42 nt | 16-31 | ORCA3 | AJ251250.1 | 687-702 | 15 of 16 | 24.3 | 1.5 | yes |
| 16 nt | 2-14 | Plastid GES | JN882025.1 | 358-346 | 12 of 13 | 18.3 | 16 | nt |
| 16 nt | 7-15 | PRX1 | AM236088.1 | 822-830 | 9 of 9 | 18.3 | 16 | no |
| 16 nt | 1-16 | STR1 | Y10182.1 | 200-215 | 16 of 16 | 32.2 | 0.001 | no |
| 42 nt | 1-42 | STR1 | Y10182.1 | 270-311 | 42 of 42 | 83.8 | 2.00E-18 | no |
| 16 nt | 2-10 | TDC | X67662.1 | 760-752 | 9 of 9 | 18.3 | 16 | yes |
| 42 nt | 12-30 | TDC | X67662.1 | 1312-1294 | 18 of 19 | 30.2 | 0.025 | yes |
| 16 nt | 2-10 | WRKY1 | KF582039.1 | 99-107 | 9 of 9 | 18.3 | 16 | yes |

**DXS2B* transcript levels also increased in the control line in response to β-estradiol, raising the possibility that the increased *DXS2B* transcript levels observed in the induced cultures of the CrBPF1-OE line were due to application of β-estradiol, rather than to increases in CrBPF1 activity.
