# Supplementary figures and images for: CrBPF1 overexpression alters transcript levels of terpenoid indole alkaloid biosynthetic and regulatory genes
Source: Front Plant Sci. 2015 Oct 1;6:818. doi: 10.3389/fpls.2015.00818 (PMC4589645; doi:10.3389/fpls.2015.00818)

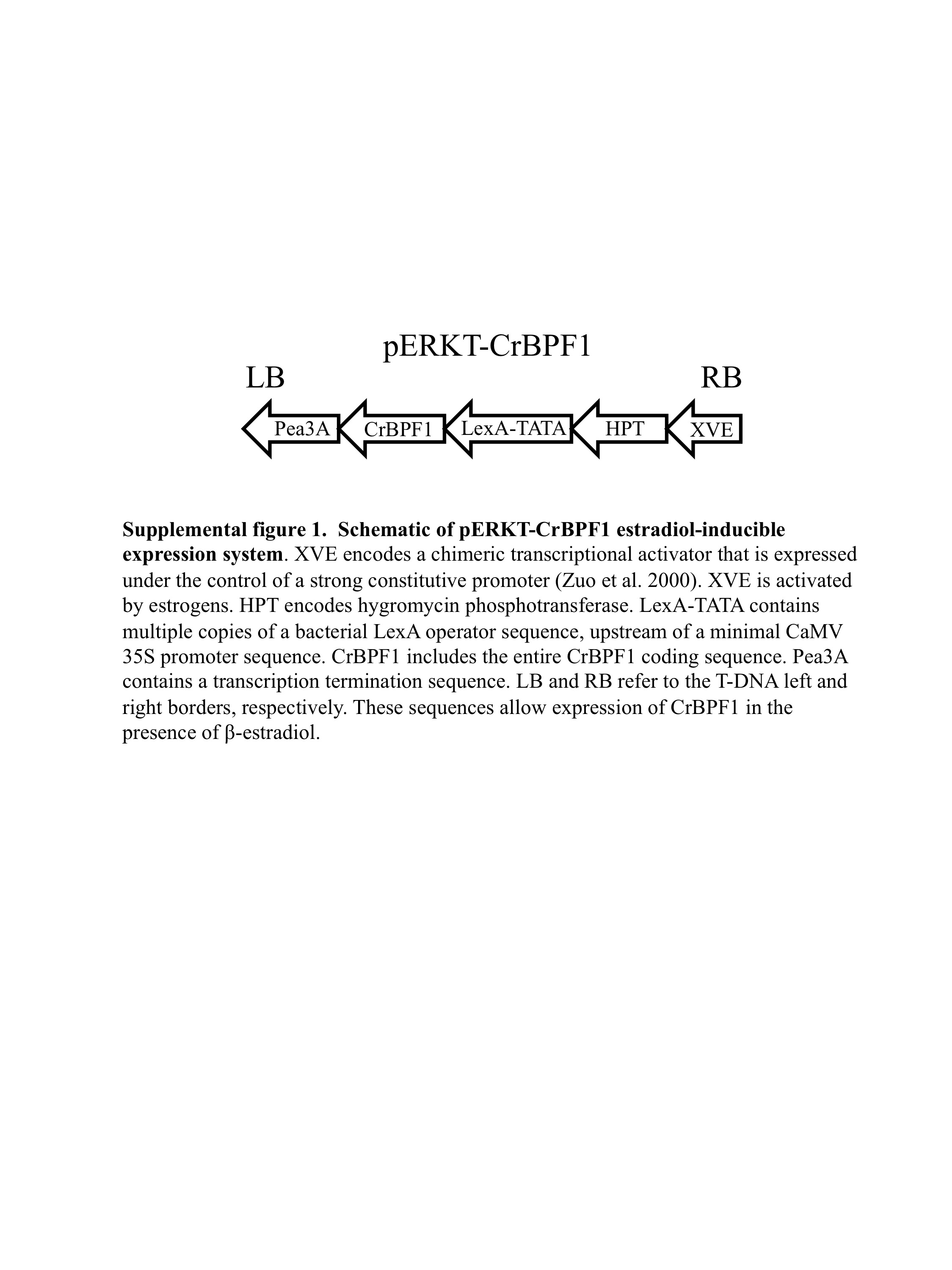

Supplement: Supplementary file 4 [file Image_1.JPEG]
